# Improving procedural skills acquisition of students during medical device training: experiments on e-Learning vs. e-Learning with hands-on

**DOI:** 10.1007/s10459-022-10148-0

**Published:** 2022-08-07

**Authors:** Tobias Grundgeiger, Franz Ertle, Daniel Diethei, Christoph Mengelkamp, Volker Held

**Affiliations:** 1 Institute Human-Computer-Media, Julius-Maximilians-Universität Würzburg, Oswald-Külpe-Weg 82, 97074 Würzburg, Germany; 2 Medical Device Management and Training, Schindeller 11, 97218 Gerbrunn, Germany; 3 Present Address: Human-Computer Interaction, Universität Bremen, Bibliothekstraße 5, 28359 Bremen, Germany

**Keywords:** Clinical skills, e-Learning, Blended learning, Education, Medical device training

## Abstract

In the context of medical device training, e-Learning can address problems like unstandardized content and different learning paces. However, staff and students value hands-on activities during medical device training. In a blended learning approach, we examined whether using a syringe pump while conducting an e-Learning program improves the procedural skills needed to operate the pump compared to using the e-Learning program only. In two experiments, the e-Learning only group learned using only the e-Learning program. The e-Learning + hands-on group was instructed to use a syringe pump during the e-Learning to repeat the presented content (section “Experiment 1”) or to alternate between learning on the e-Learning program and applying the learned content using the pump (section “Experiment 2”). We conducted a skills test, a knowledge test, and assessed confidence in using the pump immediately after learning and two weeks later. Simply repeating the content (section “Experiment 1”) did not improve performance of e-Learning + hands-on compared with e-Learning only. The instructed learning process (section “Experiment 1”) resulted in significantly better skills test performance for e-Learning + hands-on compared to the e-Learning only. Only a structured learning process based on multi-media learning principles and memory research improved procedural skills in relation to operating a medical device.

## Introduction

Insufficient training has been related to mistakes in intravenous (IV) administrations (e.g., Keers et al., 2013). Formal clinical skills training is important; otherwise, safety may depend on incidental learning from other staff (Taxis & Barber, 2003). However, medical device trainings frequently consist of classroom training with little standardization of content, no possibility for learning at an individual pace, and little hands-on activity (Brand, 2015; Grundgeiger et al., 2017; Iacovides et al., 2013; Saint-Marc et al., 2019). E-Learning, defined as technology-based learning without face-to-face contact (McCutcheon et al., 2015), can address the first points (e.g., Carolan et al., 2020; Farrell, 2006), but e-Learning in combination with concurrent hands-on activity has received little attention. The aim of the present experiments was to investigate how clinical skills acquisition can be improved by combining e-Learning and hands-on exercises in a single learning session.

E-Learning and blended learning are at least as effective as conventional learning in relation to knowledge gain (for reviews, see e.g., Bloomfield et al., 2008; Lahti et al., 2014; McCutcheon et al., 2015) and clinical skills (Li et al., 2019). However, conventional learning was frequently replaced by e-Learning or enhanced with a form of blended learning which was frequently realized by adding e-Learning to existing conventional learning (Li et al., 2019). For example, to improve the skills for IV pump use, Terry et al., (2016, 2018) compared conventional classroom training, online training with an emulated pump, and combined learning (conventional classroom training + unlimited access to the emulated pump). The combined group outperformed both classroom and online groups in a test with an actual IV pump, and there was no difference between the latter two groups. McCutcheon et al. (2015) criticized such blended learning studies because adding an e-Learning component to conventional training provides more overall learning time. Furthermore, for mandatory training sessions to legally allow staff to operate a medical product, Saint-Marc et al. (2019) suggested blended learning to combine the benefits of e-Learning and hands-on activity in a single session.

We investigate how the benefits of e-Learning may be combined with the expressed desire by staff and students for hands-on activity (Brand, 2015; Grundgeiger et al., 2017; Iacovides et al., 2013; Saint-Marc et al., 2019) in a blended learning approach for medical device training. In both experiments, one group received training with an e-Learning program and a syringe pump for hands-on practice (e-Learning + hands-on), whereas the other group used the e-Learning program only (e-Learning only). In a first session, participants conducted a 35-min training session, followed by a questionnaire with a confidence rating, a knowledge test, and a skills test. In a follow-up session approximately two weeks later, participants repeated the confidence rating, knowledge test, and skills test.

## Experiment 1

From a practical and organizational point of view, the easiest approach to incorporate hands-on activities in e-Learning is providing a syringe pump during the training. In section “Experiment 1”, we asked participants in the e-Learning + hands-on group to use the syringe pump to repeat the presented content while using the e-Learning program. In educational research, empirical studies have shown that such simple hands-on learning (e.g., a single task step is read out or demonstrated and subsequently participants repeat the task step) can be superior to conventional presentation techniques (e.g., a single task step is read out or demonstrated with no further activity) in terms of cognitive learning and retention (e.g., Hartman et al., 2000; Hearns et al., 2010; Korwin & Jones, 1990). Theoretically, the improved memory effects of hands-on learning have been explained by an additional tactile and proprioceptive experience (Vessey, 1988), or by the personal experience of success when following and independently completing step-by-step instructions (Hartman et al., 2000). In section “Experiment 1”, hands-on exercises such as opening the spring-loaded lever to hold the syringe provide additional tactile and proprioceptive experience and the personal experience of success. Due to the improved memory effects, we expected that the e-Learning + hands-on group would show better results in the skills and knowledge tests than the e-Learning only group. Due to the experience of success, we expected that the e-Learning + hands-on group would feel more confident using the syringe pump than the e-Learning only group.

### Method

#### Participants

Providing medical devices during e-Learning sessions causes an additional coordination and financial effort. We were therefore only interested in detecting a large effect of η_p_^2^ = 0.140, which is approximately a 10% difference in skills performance, for the critical between-subjects comparison of training (e-Learning + hands-on vs. e-Learning only). With a power of 1 − β = 0.80, α = 0.05, and a two tailed test, the required sample size was N = 2 × 26 participants (G*Power3, Faul et al., 2007). This research complied with the Declaration of Helsinki and was approved by the Institutional Review Board of the Institute of Human–Computer-Media. Informed consent was obtained from each participant.

**Fig. 1:**
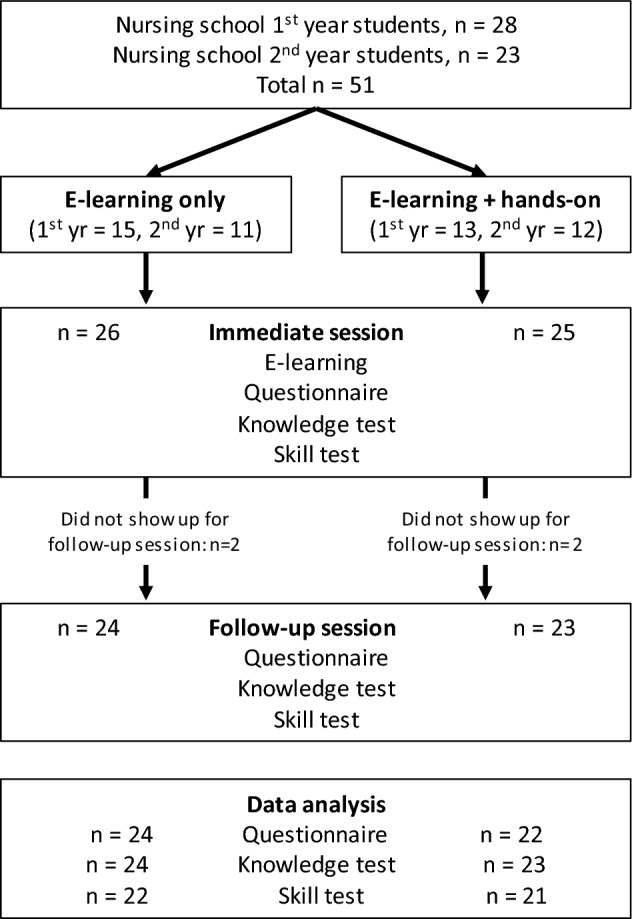
Flow diagram of design and procedure of section “Experiment 1”. Allocation of participants was random within 1st and 2nd year students. In the e-Learning + hands-on group, one participant did not answer the questionnaire. In both groups, two videos of the skills test were lost due to technical failure. yr = year

In total, 51 nursing students from a local nursing school participated, but four students did not attend the follow-up test. We included 47 participants in the analysis. Missing questionnaire responses or technical issues meant that not all dependent variables could be analyzed for all participants (Fig. 1). Mean age and gender distributions were similar in both groups (e-Learning only: *M*_*age*_ = 19.9, *SD* = 3.0, f/m = 20/4; e-Learning + hands-on: *M*_*age*_ = 19.2, *SD* = 1.8, f/m = 23/0). None of the participants had previously operated a syringe pump, and none of the participants used a syringe pump in between the immediate and the follow-up sessions.

#### Design

Participants were randomly assigned to one of two groups. The e-Learning + hands-on group was instructed to make use of a syringe pump placed next to the e-Learning computer and to repeat the presented content during the e-Learning program. The e-Learning only group used only the e-Learning program for training.

The primary dependent variable was the proportion of tasks solved in the skills test. In addition, we assessed the participants’ knowledge of operating the syringe pump, their subjective confidence in operating the pump, and the amount of assistance that they needed to solve the skills test tasks.

#### Procedure and material

The study included two standardized sessions, and there were several steps within each session (Fig. 1). First, participants provided informed consent. Participants sat down at a table with a laptop on which the e-Learning program was running. Only in the e-Learning + hands-on group was the actual syringe pump placed to the left of the laptop. The syringe pump settings (i.e., infusion rate, mechanical parts, etc.) and equipment (i.e., syringe) were set up in exactly the same way for each participant. We instructed participants in both groups to study three chapters of a syringe pump e-Learning program (see Appendix A for screenshots and further explanations). Participants were informed that each chapter had several sub-chapters. The structure was also shown in the e-Learning program and the required interaction for navigating to the different chapters and sub-chapters was demonstrated. The participants in the e-Learning + hands-on group were instructed to make use of the syringe pump and repeat the learned content while working through the e-Learning program.

The syringe pump was an Injectomat Agilia® (Fresenius Kabi Deutschland GmbH, Bad Homburg, Germany). The e-Learning program was “Medical-technical Knowledge Infusion Technology” (Version 3.0), developed by DokuPartner GmbH (Dillenburg, Germany) and the author VH in cooperation with Fresenius Kabi Deutschland GmbH. All participants were informed that the learning time was 35 min and that they were free to go through the module at their own pace. Participants were reminded of the remaining time after 15 and 30 min. All participants managed to finish the three chapters, and the hands-on activities in the case of the e-Learning + hands-on group, within the 35 min. If participants indicated that they were finished before the 35 min elapsed, the experimenter asked them to restudy the content until the full time elapsed to ensure that the training session time (i.e., learning time) was similar for both groups to avoid bias.

Second, the participants answered a questionnaire at a different table. We collected demographic data, participants’ prior knowledge of infusion pumps, and confidence in using the pump (“How confident do you feel operating the syringe pump?”; rating-scale ranging from 1—*not confident at all*—to 7—*very confident*).

Third, the participants completed a knowledge test at their own pace with no time limit. The knowledge test consisted of 14 multiple-choice questions (e.g., “How do you pause the syringe pump?”). Each question had three or four statements, and either one or more statements were correct (Grundgeiger et al., 2016).

Fourth, participants returned to the table with the syringe pump and conducted a skills test consisting of seven tasks. A teacher from the local hospital (author VH) selected the tasks to ensure that the tasks represented clinical tasks and could be solved with the learned content. Each task was printed on a card, and the stack of seven cards was placed next to the pump (see Appendix B for photo). The experimenter instructed participants to read the task, complete the task, turn the card over, and start the next task. If they were unable to complete a task, the participants could ask for help. In this case, the experimenter read out the next required sub-task from a standardized list (e.g., “press the green button”). This procedure enabled all participants to complete the task, and we documented the number of times assistance was required (see Appendix D for tasks and sub-tasks). The skills test was videotaped for later analysis.

The follow-up session was identical to the immediate session, apart from the learning session and the demographic questions.

#### Analysis

Unless indicated, we analyzed the data using a 2 (learning method) × 2 (test) mixed ANOVA using SPSS (Version 24, IBM, Chicago, IL, USA). Alpha was set to 0.05. To analyze skills performance, a task was considered correct if every sub-task was solved correctly and without assistance. We analyzed the proportion of correctly solved tasks out of the seven tasks. For knowledge, we analyzed the proportion of questions answered correctly out of the 14 questionnaire items. A single question was considered correct if every statement in relation to the question was answered correctly. To assess the amount of required assistance, we counted every sub-task for which a participant needed assistance, regardless of the amount of assistance given per sub-task.

### Results

**Fig. 2:**
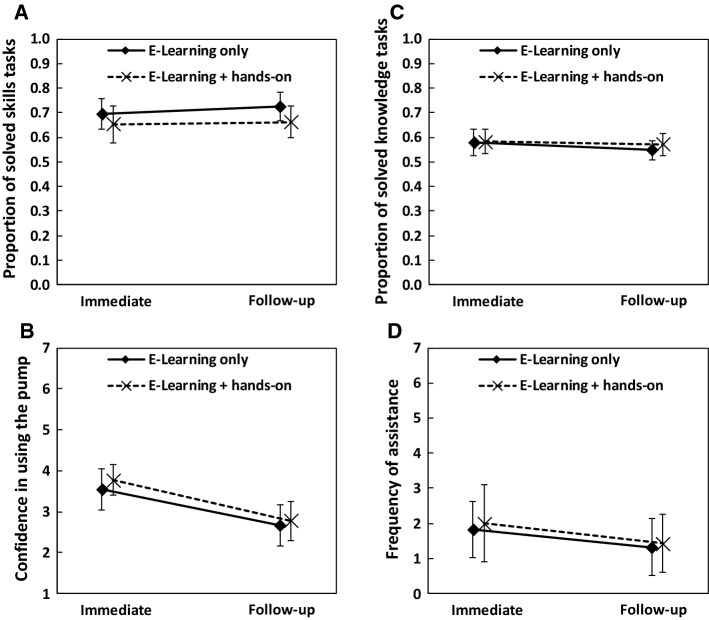
Results of section “Experiment 1”. **A** Skills test, **B** confidence in using the pump, **C** knowledge test, and **D** assistance. Bars indicate 95% CI

Considering clinical skills, the difference between the e-Learning + hands-on group and the e-Learning only group (*M* = 0.71, *SD* = 0.14) was not significant, *p* = 0.128 (Fig. 2). The factor test showed no main effect (immediate: *M* = 0.67, *SD* = 0.15 vs. follow-up: *M* = 0.69, *SD* = 0.14; *p* = 0.501). The learning method × test interaction was not significant, *p* = 0.705.

For the confidence rating, we observed no significant difference between the e-Learning + hands-on group (*M* = 3.27, *SD* = 0.97) and the e-Learning only group (*M* = 3.10, *SD* = 1.20), *p* = 0.570. A significant main effect of the factor test indicated that participants were more confident immediately after completing the e-Learning program compared to the follow-up session (immediate: *M* = 3.65, *SD* = 1.06 vs. follow-up: *M* = 2.72, *SD* = 1.10; *p* < 0.001, η_p_^2^ = 0.538). There was no learning method × test interaction, *p* = 0.635.

Considering knowledge, we observed no significant difference between the e-Learning + hands-on group (*M* = 0.58, *SD* = 0.11) and the e-Learning only group (*M* = 0.56, *SD* = 0.11), *p* = 0.598. We observed no main effect of test (immediate: *M* = 0.58, *SD* = 0.12 vs. follow-up: *M* = 0.56, *SD* = 0.10; *p* = 0.233). The learning method × test interaction was not significant, *p* = 0.593.

In relation to the number of times assistance was given, we observed no significant difference between the e-Learning + hands-on group (*M* = 1.57, *SD* = 2.11) and the e-Learning only group (*M* = 1.57, *SD* = 1.83), *p* = 0.711. We observed no main effect of test (immediate: *M* = 1.91, *SD* = 2.10 vs. follow-up: *M* = 1.37, *SD* = 1.80), *p* = 0.251. The learning method × test interaction was not significant, *p* = 0.938.

### Discussion

None of our results can support our hypotheses. Because the results of the skills test indicated a trend in the opposite direction, we conclude that the suggested training in the e-Learning + hands-on condition does not provide any benefits over e-Learning only.

There are several possible explanations for the current findings. First, participants within the e-Learning + hands-on group had to switch between the e-Learning program and the syringe pump in an unsteady manner to view the displayed information in the e-Learning program and repeat the content using the syringe pump. Considering the so-called split-attention effect (e.g., Ayres & Sweller, 2014), switching back and forth might have increased the participants’ cognitive extraneous load (e.g., Sweller et al., 2019). The arrangement of the learning environment may have increased an already considerable cognitive load and reduced the cognitive load available for actual learning. Indeed, at the end of the session, several of the students complained informally about having to use both the pump and the e-Learning program simultaneously.

Second, the instruction to use both the e-Learning program and the syringe pump may not have been sufficiently clear. However, based on the observations of the experimenters, all participants used the pump to apply the presented information, and only a few participants did not use the pump extensively. Furthermore, the videos and pictures that were part of the e-Learning might have produced an illusion of knowledge (Serra & Dunlosky, 2010) leading to the feeling that there was no need to spend much attention on practicing the procedures using the syringe pump.

Third, although using the pump requires participants to apply the learned content, the learning environment may be improved in terms of how the learned content has to be retrieved from memory (Eiriksdottir & Catrambone, 2011). Researchers have shown that simply replicating the learned content is less efficient than elaborative learning, such as actively recalling the content without access to the learned material (Karpicke & Blunt, 2011).

## Experiment 2

In section “Experiment 2”, we implemented an elaborative learning procedure by dividing the learning phase into short e-Learning units followed by an associated hands-on exercise. The exercise was conducted separately from the e-Learning program to enhance active recall of the content (Karpicke & Blunt, 2011). This training procedure also solves the issue of having to split one’s attention (Ayres & Sweller, 2014) between the e-Learning program and the syringe pump and may leave more cognitive resources available for learning the content. We expected that the e-Learning + hands-on group would show better results in the knowledge and skills tests and that they would feel more confident using the syringe pump compared to e-Learning only.

### Method

Because the methods of section “Experiment 2” were based on section “Experiment 1”, we report only deviations from section “Experiment 1”.

#### Participants

**Fig. 3:**
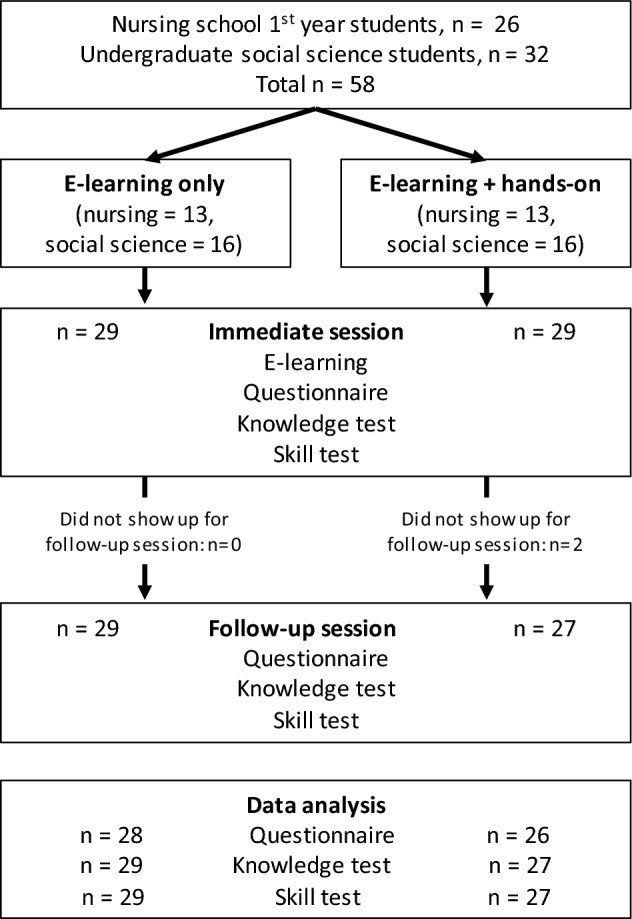
Flow diagram of design and procedure of section “Experiment 2”. Allocation of participants was random within nursing and social science students. In both groups, one participant did not answer the questionnaire

Due to the limited availability of nursing students, we also included social science undergraduate students. In total, 26 nursing students and 32 undergraduate students participated, but two students did not show up for the follow-up test. We included 56 participants in the analysis (Fig. 3). Mean age distributions were similar in both groups (e-Learning only: *M*_*age*_ = 20.9, *SD* = 2.9; e-Learning + hands-on: *M*_*age*_ = 20.1, *SD* = 2.7). However, despite randomization, the gender distribution was significantly different (e-Learning only: f/m = 17/12; e-Learning + hands-on: f/m = 23/4; Fisher’s exact test, *p* = 0.038). We considered gender and cohort (nursing vs. social science) in the initial analysis, but because both factors did not affect the critical factor learning method, we summarized these results in a supplementary analysis (Appendix D). Two of the nursing students indicated that they operated a syringe pump under supervision before, and none of the participants used a syringe pump in between the immediate and the follow-up sessions.

#### Procedure and material

Data collection took place in a separate, quiet room in the hospital or at the university. For the participants in the e-Learning + hands-on group, the content was structured in six units (Appendix E). After each unit, participants were instructed to apply the learned content. Participants were provided with a syringe pump that was placed on a table behind them. In this setup, participants had to actively retrieve the content from memory during the hands-on part because they could not see or interact with the e-Learning at the same time. If participants could not remember the next step to accomplish the task, they were allowed to return to the e-Learning program to look up the missing information.

In a pilot study (*n* = 4), we recorded the required time for each of the six e-Learning units and hands-on parts. We used the mean times to time the e-Learning units and hands-on parts. We played a sound to indicate when the participants should proceed with the next step (i.e., stop the e-Learning unit and start the hands-on part, stop the hands-on part and start the next e-Learning unit, etc.). If participants finished a step early, they were instructed to revise the content (or apply the content in the case of the e-Learning + hands-on group).

The participants in the e-Learning only group were instructed to study the six e-Learning units, and a sound indicated when to proceed to the next unit. The study time for the participants in the e-Learning only group for each unit was equal to the study time of the e-Learning + hands-on group for each unit and subsequent exercise.

We did not interrupt participants if the time limit for each e-Learning unit or hands-on part ended. Instead, we measured the additional time taken if participants took more than 10 s to proceed to the next unit or hands-on part after the sound was played.

### Results

**Fig. 4:**
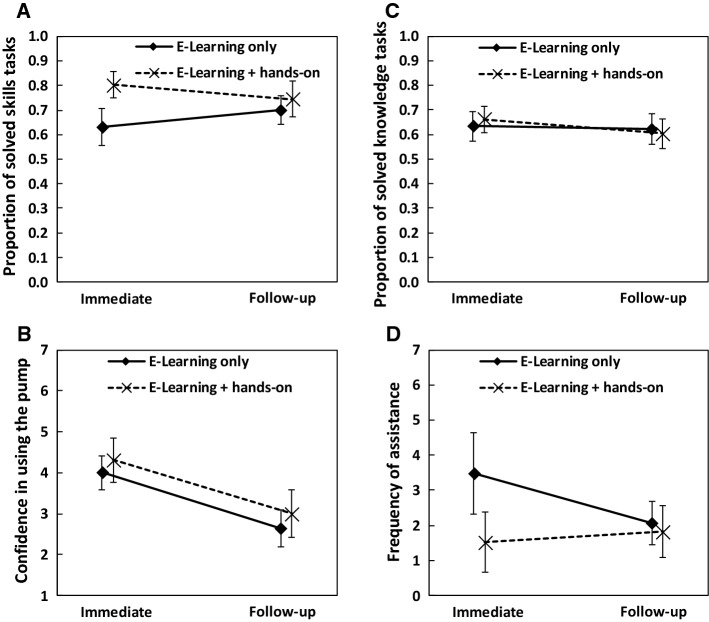
Results of section “Experiment 2”. **A** Skills test, **B** confidence in using the pump, **C** knowledge test, and **D** assistance. Bars indicate 95% CI

The e-Learning + hands-on group (*M* = 0.77, *SD* = 0.16) solved significantly more tasks in the skills test than the e-Learning only group (*M* = 0.67, *SD* = 0.18), *p* = 0.008, η_p_^2^ = 0.124 (Fig. 4). The factor test showed no main effect (immediate: *M* = 0.71, *SD* = 0.19 vs. follow-up: *M* = 0.72, *SD* = 0.17; *p* = 0.813). The learning method × test interaction was significant, *p* = 0.007, η_p_^2^ = 0.128. Bonferroni-corrected t-tests (alpha = 0.025) showed that the better performance of the e-Learning + hands-on group (*M* = 0.80, *SD* = 0.13) compared to the e-Learning only group (*M* = 0.63, *SD* = 0.20) is mostly due to significant benefits in the immediate session, *p* < 0.001, *d* = 1.01. We observed no significant difference in the follow-up session, *p* = 0.327 (e-Learning + hands-on: *M* = 0.74, *SD* = 0.18; e-Learning only: *M* = 0.70, *SD* = 0.15).

For the confidence rating, we observed no significant difference between the e-Learning + hands-on group (*M* = 3.65, *SD* = 1.41) and the e-Learning only group (*M* = 3.32, *SD* = 1.11), *p* = 0.249. A significant main effect of the factor test indicated that the participants were more confident immediately after completing the e-Learning program compared to in the follow-up session (immediate: *M* = 4.15, *SD* = 1.20 vs. follow-up: *M* = 2.81, *SD* = 1.31; *p* < 0.001; η_p_^2^ = 0.479). There was no learning method × test interaction, *p* = 0.898.

We observed no significant difference between the e-Learning + hands-on group (*M* = 0.63, *SD* = 0.15) and the e-Learning only group (*M* = 0.63, *SD* = 0.16) in the knowledge test, *p* = 0.914. Participants performed significantly better in the immediate session (*M* = 0.65, *SD* = 0.15) than in the follow-up session (*M* = 0.61, *SD* = 0.16), *p* = 0.031, η_p_^2^ = 0.083. The learning method × test interaction was not significant, *p* = 0.121.

Participants in the e-Learning + hands-on group (*M* = 1.67, *SD* = 2.01) required significantly less assistance than participants in the e-Learning only group (*M* = 2.78, *SD* = 2.31), *p* = 0.028, η_p_^2^ = 0.087. We observed no main effect of test (immediate: *M* = 2.54, *SD* = 2.99 vs. follow-up: *M* = 1.95, *SD* = 1.73; *p* = 0.107). The learning method × test interaction was significant, *p* = 0.015, η_p_^2^ = 0.104. Bonferroni-corrected t-tests (alpha = 0.025) showed that the e-Learning + hands-on group (*M* = 1.52, *SD* = 2.14) required significantly less help than the e-Learning only group (*M* = 3.48, *SD* = 3.02) in the immediate session, *p* = 0.007, *d* = 0.75. We observed no significant difference in the follow-up session (e-Learning + hands-on group: *M* = 1.81, *SD* = 1.88, e-Learning only: *M* = 2.07, *SD* = 1.60), *p* = 0.588.

In the e-Learning + hands-on group, 19 participants took additional time, and the mean accumulated additional learning time per unit was *M* = 12 s (*SD* = 12 s). In the e-Learning only group, eight participants took additional time, and the mean time was *M* = 14 s (*SD* = 9 s). A Mann–Whitney U test showed no significant difference (*n* = 27, *U* = 54, *p* = 0.260). Furthermore, none of our dependent variables correlated with the mean accumulated additional learning time.

### Discussion

As expected, the e-Learning + hands-on group outperformed the e-Learning only group in the skills test (main effect of learning method). Although the learning method × test interaction indicated that the benefits of hands-on training were smaller and no longer statistically significant in the follow-up session, the e-Learning + hands-on group still showed a descriptively better performance. We observed a descriptive but non-significant (*p* = 0.060) improved performance in the e-Learning only group in the follow-up skills test compared to the immediate test. This can be explained by the additional learning opportunity due to more assistance during the immediate skills test. Such an interpretation is supported by the learning method × test interaction for the number of times assistance was given. The e-Learning only group needed significantly more assistance than the e-Learning + hands-on group in the immediate session, but not in the follow-up session. This assistance is a form of elaborated feedback (i.e., feedback providing an explanation) and adaptive scaffolding (i.e., helping students when they are unable to perform a task on their own), which are effective tools to support multimedia learning (for a meta-analysis on adaptive scaffolding, see Belland et al., 2017; on elaborated feedback, see Van der Kleij et al., 2015). Therefore, it seems reasonable that our procedure in the skills test promoted learning and thus explains why the e-Learning only group was better in the follow-up test than in the immediate test.

Contrary to our hypothesis and despite better skills test performance, the e-Learning + hands-on group did not feel more confident in using the pump. One reason may be that the confidence rating was conducted before the skills test. Perhaps the e-Learning + hands-on group would have felt more confident after having a positive experience of self-efficacy during the skills test.

We did not observe the hypothesized benefits for knowledge gain. Although it has been reported that hands-on learning can improve cognitive learning (Hearns et al., 2010), the learned content and the presentation of the content between the groups in the present study were the same. It is therefore not too surprising that the learned knowledge did not differ between the groups.

## General discussion and conclusion

In the present experiments, specific instructions on how to combine e-Learning and hands-on units improved clinical skills to operate a syringe pump (section “Experiment 2”). However, simply providing a medical device and instructing trainees to make use of the device to repeat the presented information during an e-Learning training session did not improve clinical skills (section “Experiment 1”). The present results do not support the idea that the improved memory effects of hands-on training were due to additional tactile and proprioceptive experience or the personal experience of success when completing a task (Hartman et al., 2000). If this had been the case, we should have observed a benefit of hands-on learning when simply providing the syringe pump (section “Experiment 1”). However, the procedure of Hartman et al. (2000) incidentally included a division of learning and exercise during the learning phase and also included active recall for the exercise. The split attention principle (Ayres & Sweller, 2014) and active memory retrieval during learning (Karpicke & Blunt, 2011) can therefore explain Hartman et al.’s (2000) results and the results of the present study.

In addition, further memory effects may have contributed to the effect in section “Experiment 2” or may be considered in future blended learning approaches. Transfer appropriate processing (Morris et al., 1977) suggests that the learning activity must be defined to a goal and that encoding (i.e., learning) and retrieval (i.e., skills test or using a medical device in the actual context) use the same memory processes; therefore, one should observe better performance if the process of retrieval matches the process of encoding. A further and related memory effect is the encoding specificity principle (Tulving & Thomson, 1973), which suggests that a match of encoding context and retrieval context results in increased recall compared to a change in context. Since we did not observe an effect of learning method in section “Experiment 1”, we consider it unlikely that the effect of learning method in the skills test in section “Experiment 2” was solely caused by either of the above memory effects. From an applied perspective, however, it would make sense to run training in a similar physical environment and using the same memory processes as in the users’ actual work contexts. From a research perspective, distinguishing the single mechanism that caused the effect in section “Experiment 2” is a challenging but interesting and valuable endeavor, and it could provide practitioners with better support for teaching and training regarding the use of medical devices or other clinical tasks.

Our study has several limitations. First, due to limited availability of nursing students, we also included social science university students, who are not representative of the target population. However, the supplementary analysis (Appendix D) showed that the factor cohort did not affect the critical comparison of learning method (e-Learning + hands-on vs. e-Learning only). Second, we used only a single and a rather elaborate e-Learning program, and thus one may question the translation of our results to other e-Learning programs. Third, our focus was on procedural knowledge and we did not evaluate other aspects of clinical skills, such as basic science knowledge and clinical reasoning (cf. Michels et al., 2012). Fourth, our experiments remain unclear about the exact theoretical mechanism that caused the improved skills performance in the e-Learning + hands-on group. Fifth, we did not apply a pre-test to assess the skills and knowledge of the nursing and social science students in using syringe pumps. However, only two out of all 103 participants indicated to have operated a syringe pump under supervision before participating in the study.

E-Learning can be at least as effective as conventional learning (e.g., Lahti et al., 2014; Li et al., 2019; McCutcheon et al., 2015). The present results indicate that pairing e-Learning with hands-on exercises can further enhance skill learning. However, our results suggest that trainees need clear instructions on how to use the device in combination with the e-Learning program; providing a syringe pump and leaving it up to the learners to decide how to use it is insufficient. The necessary instructions could be provided to students by a teacher or to trainees by a medical device trainer. Another possibility could be to implement prompts in e-Learning programs after each section to tell trainees to apply the knowledge before beginning the next section. However, the suggested learning and exercise process also requires the trainee to understand and comply with the training method. Alternatively, trainees may self-regulate their training process; in other words, they need to judge their knowledge and skills accurately to decide whether they need hands-on training using the syringe pump or whether they just need to rehearse parts of the e-Learning program. To our knowledge, self-regulation and metacognition (de Bruin & van Merriënboer, 2017) are still understudied aspects in medical device training.

A final practical aspect is that most learners do not want to give up on conventional learning methods (e.g., Bloomfield & Cornish, 2015) and hands-on activities (Saint-Marc et al., 2019). Including the training method of the present study in classroom teaching may be a fruitful way to combine the wish for hands-on activities by trainees and the benefits of e-Learning, such as individually paced learning and standardized content, while maintaining the benefits of having a teacher or trainer at hand when needed and providing the opportunity for hands-on activities.

## Appendices

## Appendix A: Screenshots of e-Learning program

Note: Participants had to learn the chapters “Intro”, “Interaktives Training” (interactive training), and “Menü Funktionen” (menu functions) of the menu on the gray horizontal bar.

See Appendix Figs. 5, 6, and 7.

**Fig. 5:**
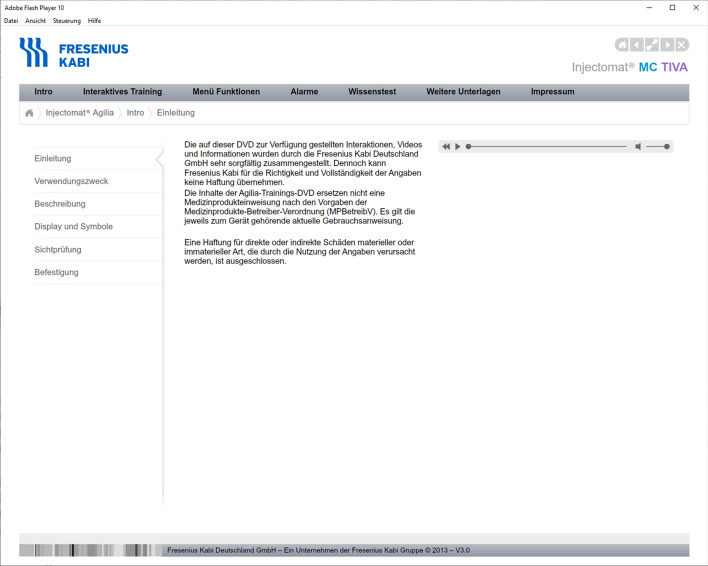
Screenshot of the Chapter “Intro”. The menu on the left shows the sub-chapters. The sub-chapter “Einleitung” (Introduction) is shown. An audio of the text can be played back with the audio controller on the right. The content of the chapter “Intro” includes legal matters, a general description of the pump, details on the visual inspection before use, and mounting the device to, for example, a pole

**Fig. 6:**
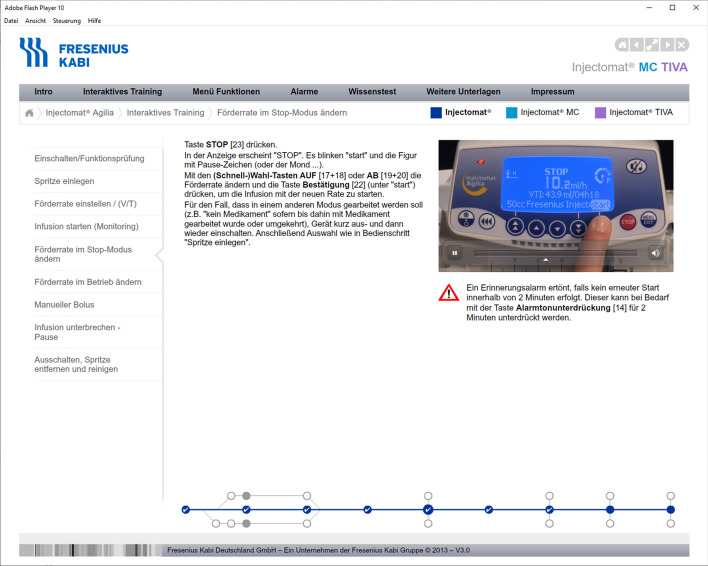
Screenshot of the Chapter “Interaktives Training” (interactive training). The sub-chapter “Förderrate im Stop-Modus ändern” (changing the infusion rate while the pump function is paused) is shown. The video shows how to operate the pump. The audio of the video and the text on the left of the video are identical. The note below the video highlights an alarm that could occur if the pump is not restarted. The diagram at the bottom works like a progress bar for the current chapter. The content of the chapter “Interaktives Training” (interactive training) includes switching the pump on and functional testing, inserting the syringe, setting the infusion rate, starting/stopping the infusion, changing the infusion rate, manual bolus, pause the infusion, and switching the device off

**Fig. 7:**
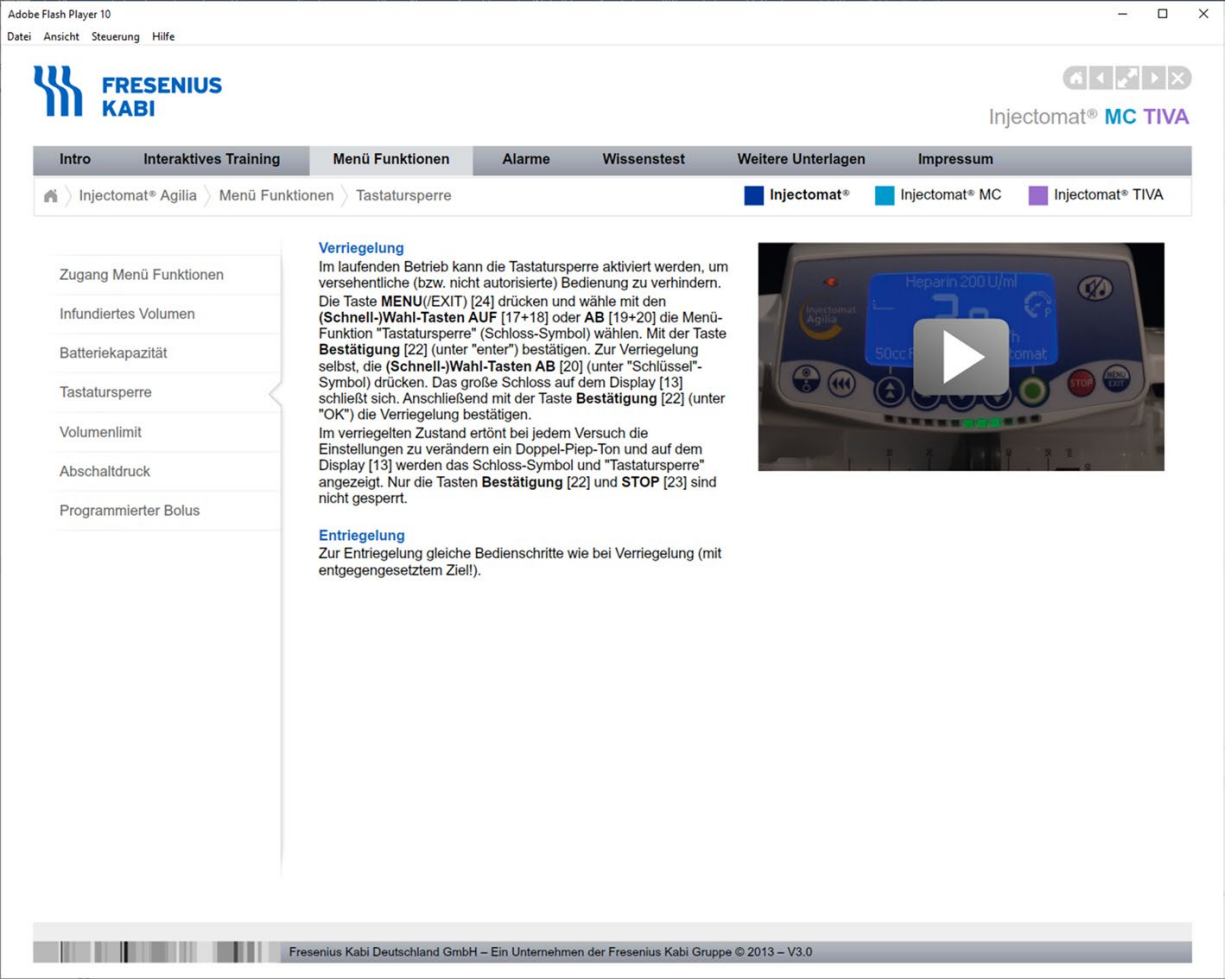
Screenshot of the Chapter “Menü Funktionen” (menu functions). The sub-chapter “Tastatursperre” (keyboard lock) is shown. The video shows how to operate the pump. The audio of the video and the text on the left of the video are identical. The content of the chapter “Menü Funktionen (menu functions) includes menu access, infused volumes, battery capacity, key lock, volume limit, cutout pressure, and bolus programming

## Appendix B: Photo of skills test

See Appendix Fig. 8.

**Fig. 8:**
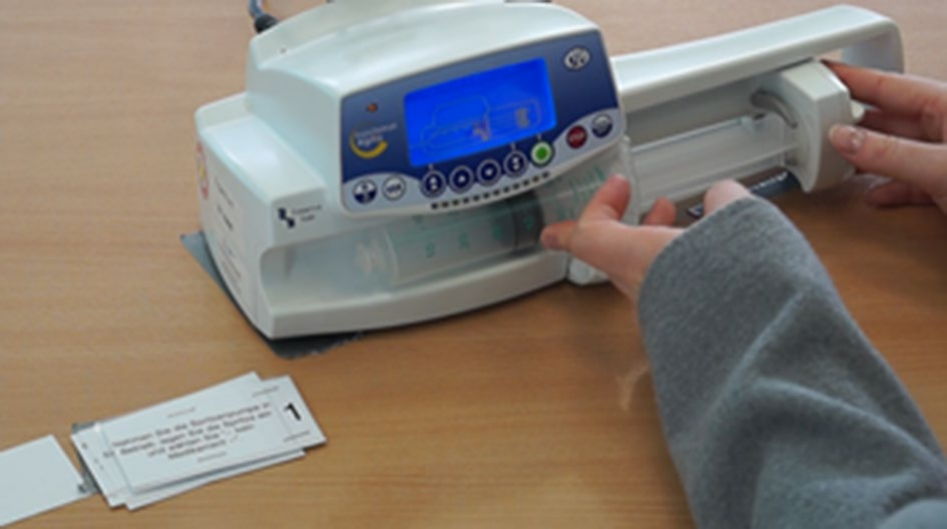
Screenshot of the Video Analysis During the Skills Test. On the left, the current task (i.e., task number 1: switch on syringe pump, insert the syringe, and choose “—no medication—”) is displayed on a card. The location of the syringe pump was marked with tape on the table to ensure that the video captured all relevant actions

## Appendix C: The seven skill test tasks

Note: The text after the colon shows the printed instructions on the seven cards (original in German). The experimenter provided a sub-take verbally (i.e., a., b., etc.) if a participant required assistance. For section “Experiment 2”, the order of tasks 4 and 5 were switched.

Task 1: Switch on syringe pump, insert the syringe, and choose “—no medication—”

- Press power button on left side of pump.
- Move syringe driver to right.
- Insert syringe with label facing front.
- Move driver to the left. The lever needs to properly enclose the forcer of the syringe.
- Confirm first menu entry “—no medication—” with green button.

Task 2: Set the rate to 5 ml/hr

- Push speed dial keys up or down to select the infusion rate of 5 ml/hr.

Task 3: Set the rate to infuse 20 ml within the next 3 h. Do not set a rate to keep the vein open.

- Push menu button
- Push speed dial keys up or down to select menu point “V/T”.
- Press green button.
- Push speed dial keys up or down to select a volume of 20 ml.
- Press green button.
- Push speed dial keys up or down to set the time for 3 h.
- Press green button four times.

Task 4: Now set a volume limit of 2 ml. If the limit is reached, the rate of 2 ml/hr should be set to keep the vein open

- Push menu button.
- Push speed dial keys up or down to select menu point “V/T“.
- Press green button.
- Push speed dial keys up or down to select a maximum volume of 20 ml.
- Press green button.
- Push speed dial keys up or down to select the rate to keep the vain open and set it to 2 ml/hr.
- Press green button.
- Press menu/exit.

Task 5: Administer a bolus of 2.5 ml with a rate of 900 ml/hr

- Press the bolus button.
- Press the bolus button until the bolus rate starts to blink.
- Push speed dial keys up or down to set the bolus rate to 900 ml/hr.
- Press green button.
- Press the bolus button.
- Within two seconds, press the bolus button again.
- Keep the bolus button pressed until the rate is infused.

Task 6: Set a pause of 2 h and 30 min

Option 1:

- Press the stop button twice.
- Push speed dial keys up or down to set 2 h.
- Press green button.
- Push speed dial keys up or down to set 30 min.
- Press green button.

Option 2:

- Push the pause button.
- Push menu/exit button.
- Push speed dial keys up or down and select the pause function.
- Press green button.
- Push speed dial keys up or down to set 2 h.
- Press green button.
- Push speed dial keys up or down to set 2 h.
- Press green button.

Task 7: Switch off the syringe pump according to the specifications

- Push power button until the displays turns dark.

## Appendix D: Gender and cohort analyses and discussion

Besides the 2 (learning method) × 2 (test) mixed ANOVAs, we repeated each test with the additional factor cohort (nursing vs. social science) or gender (male vs. female). Because the factors cohort and gender never interacted with the critical factor learning method, only a brief summary is given of the significant effects of both factors.

Social science students solved more tasks than nursing students (main effect of the cohort, *p* = 0.021). The difference between the cohorts was smaller in the follow-up session compared to the immediate session because the nursing students showed improved performance in the follow-up test compared to the immediate test (cohort × test interaction, *p* = 0.006). Finally, male participants solved more tasks than female participants did (main effect of the gender, *p* = 0.004) but, critically, gender did not interact with any other factor.

Social science students required fewer instances of assistance than nursing students (main effect of the cohort, *p* = 0.005). The difference between the cohorts was smaller in the follow-up session compared to the immediate session because the nursing students required less assistance in the follow-up test than in the immediate test (cohort × test interaction, *p* = 0.028).

As in a similar study (Grundgeiger et al., 2016), we observed no differences between the cohorts in relation to the knowledge test. We did observe a better skills test performance by the social science students than the nursing students in the immediate test, but not in the follow-up test (cohort × test interaction). Similar to the learning method × test interaction (see main text discussion of section “Experiment 2”), the number of times assistance was required can explain the cohort skills performance result pattern. Nursing students required more assistance in the immediate test, but not in the follow-up test (cohort × test interaction). The assistances in the immediate test were an additional learning opportunity and therefore the nursing students required less assistances in the follow-up test but showed improved skills task performance in the follow-up test. The better skills task performance of the social science students may be explained by their higher education level of who all had an A-level degree (13 year of school education). Nursing is not a university degree in Germany and only 38% had an A-level degree and 62% secondary school degrees (10 years of school education). However, if this was the main reason, one may also expect to observe better knowledge task performance. Finally, and most importantly, the critical comparison of the learning method was not affected by the cohort or gender.

## Appendix E: The content of the six learning units in section “Experiment 2”

1. Introduction (whole content), interactive training (switching on/ functional test, insert syringe)
2. Interactive training (setting the infusion rate, starting/stopping the infusion)
3. Interactive training (changing the infusion rate)
4. Interactive training (manual bolus)
5. Menu (whole content)
6. Interactive training (pause, switch off pump)
